# The Early Days of Ovariotomy1Read at meeting of the Canadian Medical Association, London, Ont., August 26, 1903; also printed in the *Canadian Jour. Med. and Surg.*, September, 1903.

**Published:** 1904-03

**Authors:** Matthew D. Mann

**Affiliations:** Buffalo, N. Y.


### Buffalo Medical Journal.

Vol. XLIII.—LIX. MARCH, 1904. No. 8.

### ORIGINAL COMMUNICATIONS.

### The Early Days of Ovariotomy.[1]

By MATTHEW D. MANN, A. M., M. D., Buffalo, N. Y.

THE rising generation, which has only seen abdominal surgery in its full development, is apt to forget the trials and struggles of those who first attempted to open the abdomen, and who finally put the operation on a firm basis. Few can realise now the amount of opposition, both within and without the profession, which existed. McDowell, as we shall hear, was threatened with death; and later operators were almost ostracised for attempting this "murderous operation." In the last twenty years, the triumphs of surgery have been so great that now no operation, no matter what its magnitude, is condemned untried, and the result is awaited with patient and indulgent expectation. But only forty years ago, this was not so, and at the time that abdominal surgery had its beginning, the feeling of opposition to "butchering," as they called it, was most emphatic and unreasoning.

Abdominal surgery had its beginning in America. Many attempts have been made to wrest this triumph from us, but all have failed. The claims of the United States are now generally admitted as being clearly proved, and the name of the first operator rescued from oblivion and duly honored.

The first abdominal section, having for its object the removal of an ovarion tumor, was done by Dr. Ephraim McDowell, on December 13, 1809, in Danville, Ky. Although practising in what was then the backwoods, McDowell was by no means an uneducated, ignorant, or pretentious adventurer. The operation was done after long consideration, after a full understanding of

1. Read at meeting of the Canadian Medical Association, London, Ont., August 26, 1903; also printed in the *Canadian Jour. Med. and Surg.*, September, 1903.

the difficulties with which he had to contend, and a careful planning of the technique. He had been a student of the great John Bell, in Edinburgh, and while there had heard it suggested that perhaps an ovarian tumor could be successfully removed. He formed the determination at that time that, if the proper case ever presented itself, he would make the effort to operate. After his return to Danville, he was sent for to see a Mrs. Crawford, residing a long distance away. McDowell found her trouble to be an ovarian tumor, and gave a fatal prognosis unless she was relieved by the knife. To quote from Dr. Gross:

After a most thorough and critical examination, Dr. McDowell informed his patient, a woman of unusual courage and strength of mind, that the only chance for relief was the removal of the diseased mass. He explained to her, with great clearness and fidelity, the nature and hazard of the operation. He told her that he had never performed it, but that he was ready, if she were willing, to undertake it, and to risk his reputation on the issue, adding that it was an experiment, but one well worthy of trial.

Mrs. Crawford accepted the opinion of the physician with great coolness, and promptly assured him that she was not only willing, but ready to submit to his decision, asserting that any hope of relief was preferable to the agony she suffered. She traveled on horseback—the only mode of locomotion in those days—to the home of Dr. McDowell, sixty miles away. So great was the weight of the tumor resting upon the pommel of the saddle, that a large contusion was formed on the skin.

On the day of the operation, McDowell was conscious that an angry and excited mob of men had collected outside of his house, openly threatening to hang him if his experiment of "butchering a woman" did not succeed. There is no doubt that if the woman had died, McDowell would have lost his life at the hands of his infuriated townsmen.

I have often wondered which was the braver—the man or the woman—the woman, to subject herself to an operation which she knew had never been done, an experiment which would cause intense suffering at the time, anesthetics being unknown, and the result of which must be uncertain; the man, to risk his life for the mere sake of doing good, without hope of reward, except, perhaps, a modest fee, and with certain death confronting him if he failed. It seems to me that the bravery of the man was the greater. He put his life at stake without any necessity impelling him, except his love of humanity and his desire to do good; while the woman had death staring her in the face, and was accepting an opportunity which had never yet been offered to anybody, to escape the terrible, persistent suffering which would certainly come. To quote from Thomas Keith: "She had not much to lose—a few months only, it may be, of ever-increasing suffering—and she might gain much by an operation, having much to gain." Fortunately for the good of mankind, and of womankind in particular the operation was successful.

The technique of the operation sounds a good deal like an operation done today. The incision was made, about nine inches long, a little to the left of the median line. The tumor was then opened, its contents allowed to escape; after which it was removed from the abdomen, the pedicle tied by strong silk ligature, and the tumor cut off. After this the patient was turned upon her side, to allow all the blood and fluid to escape. This having been accomplished, she was turned on her back, the intestines replaced, and the wound closed by an interrupted suture, the ligature hanging out of the lower end of the incision. Dressings were applied, and the patient put to bed. Five days later, McDowell, on visiting her, found her making her bed. In twenty-five days she returned home in good health, and lived for thirty-two years after, she having been forty-seven at the time of the operation.

McDowell afterwards operated on twelve cases, eight of the thirteen being successful—a record which was not beaten until the advent of antiseptic surgery.

McDowell is described as a tall, strikingly handsome man, with an erect and commanding figure and lustrous black eyes, which seemed to penetrate the very thoughts of those who looked into them. His refinement and intellectual powers were of the highest type. Many stories are told illustrative of his abilities of mind: his unflinching adherence to duty in the face of adversity and difficulties seems to have been one of his strongest points. Stories are told of his adventurous rides through the woods, of fording rushing torrents filled with ice and driftwood, and other anecdotes which illustrate the nobility and force of the man's character. He might well have stood for the original of MacLeod, Ian MacLaren's justly famous hero.

McDowell was a man of strong religious convictions, and we have left to us a very forcible petition offered by him to Almighty God, a few hours before the appointed time to make the first ovariotomy. Who will say that it was not in answer to this prayer that his hand was guided to bring to a successful termination his momentous and trying experiment, fraught with interest, not only to the operator, but to humanity? It was certainly a trying hour to him, and we can well understand that he should have asked for strength and guidance where he thought he could best obtain them. His biographer says: "His abiding faith in the efficacy of prayer was beautiful, and no doubt his remarkable success in the field of surgery can be largely attributed to his strong convictions and unwavering faith in the Great Jehovah."

After McDowell, no operations of this kind were done until 1821, when Dr. Nathan Smith, Professor of Surgery in Yale College, performed a successful ovariotomy. He was just as much entitled to the honors of a discoverer as was McDowell, for he had never heard of the Kentucky surgeon or of this operation. His methods were different, but the result was just as good.

The third successful ovariotomist was Dr. Alban C. Smith, of Danville, who had been a partner of McDowell's. He operated in 1823. A few scattering operations were done after that, but it was not until 1843-44 that a new impulse was given by the success of Dr. John L. Atlee, which was still further aided by his brother, Dr. Washington L. Atlee, of Pennsylvania. After this, cases became more common, and, taking the country at large, several were reported every year, until in 1855, there were twenty-one cases, with six successes and fifteen deaths. This heavy mortality seems to have had the effect of diminishing the number, as they fell off rapidly, until in the years 1860-63, there were only three in each year. In 1870, Dr. Atlee reported his 200th case, while Kimball had had 121, and Dunlap, Peaslee, J. P. White, McRuer, Thomas, Bradford, Emmet, and Sims had had from 60 to 12 cases each.

In England the operators who first made reputations were Tyler Smith, Baker Brown, Chas. Clay, Thos. Bryant, Thomas Keith, and Spencer Wells. To the latter we must unquestionably give the credit of having contributed largely to influence the profession, and to overcome the opposition which, up to 1860, had existed in England more than anywhere else. Many prominent men opposed the operation, very broadly denouncing those who attempted it as murderers, as guilty of malpractice, and using all their influence to keep the operation down. After Sir Spencer Wells's paper in 1860, opposition was silenced, and from that date it may be said that ovariotomy was adopted as a legitimate resource in England.

My own experience of ovariotomy began in 1870, when I entered the Strangers' Hospital, in New York city, as interne. Dr. T. G. Thomas was appointed gynecologist to this hospital, which had just been established; and, filled with ardor and enthusiasm, he soon collected a considerable number of cases for operation. During the year that I served as senior assistant and house-surgeon, I had under my care twelve operation cases, nine of which recovered. As can be readily imagined, an ovariotomy in those days was a great event. I have seen in the operating room at the hospital, witnessing and advising, and perhaps assisting, Drs. Thomas, Sims, Peaslee, Emmet, Noeggerath, Sands, Willard Parker, and others of the great lights of surgery in New York at that time. As we had no trained nurses, Dr. E. L. Trudeau, who was my senior by six months, and myself had to take the entire charge of the cases. The nurse would call us frequently during the night, and we would pass the catheter, give hypodermics of morphine, and do all the nursing which is now so much better done by our skilled and trained assistants.

Dr. Thomas's theory in those days was that a great deal of the danger was due to the shock to the nervous system, which led to inflammation; and in order to quiet the nervous system, the patient was put under the influence of opium for a few days in advance of the operation. We can see here the influence of the Alonzo-Clark treatment of peritonitis: if large doses of opium would cure peritonitis, smaller doses would prevent. And so, in order to head off the disease, of which everybody stood in holy terror, the opium was given before the operation was commenced.

Dr. Peaslee was the first to perform drainage, which he did as early as 1855. He passed a catheter through the vaginal wall into Douglas's cul-de-sac at the time of an operation, and left it there, corking the end. Septic symptoms supervening, he removed the cork, and allowed the fluid to come away, and followed it by copious injections into the peritoneal cavity of salt solution, and later by a weak solution of chlorinated soda. He published a paper on the subject in 1870. Thomas immediately took up the idea, following Peaslee's plan of putting a linen tent into the lower angle of the wound. Soon after this the idea of a drainage tube came from Koeberle of Germany. Thomas immediately began its use.

I remember very well the first drainage tube (1871), which was an old-fashioned, hard-rubber vaginal syringe, an inch in diameter, with four holes at the round end. This was introduced on the second day, the tent of cloth which had been placed in the lower angle of the wound the day of the operation, being removed.

Dr. Thomas also followed Peaslee by washing out the abdomen in a septic case, after the operation, using a solution of hyposulphite of soda. As early as 1871, he washed out the abdomen before closing the wound. Antiseptic ideas were then just beginning to dawn. Carbolic acid had just been discovered, and Lister was making his first experiments in what we now call "Listerism," experiments which were destined to revolutionise surgical methods, and to make the name of Sir Joseph Lister one of the greatest in the record of the benefactors of the race.

Although, as already mentioned, drainage was used before Sims began to do abdominal work, it was his paper, published in 1872, which really popularised drainage in abdominal cases.

Dr. Thomas, up to 1870, had had twenty-seven ovariotomies, and was only excelled by one other operator in New York, namely, Dr. Peaslee, who had had twenty-eight. Sims, who never made a great name as an abdominal surgeon, had had only twelve. It must be remembered that at this time all other forms of abdominal surgery were unknown and almost undreamed of. I remember very well when Pean's book came out, about 1871, detailing the histories of a large number of fibroids that had been successfully removed, that Dr. Thomas expressed very grave doubt as to the truthfulness of the histories.

In those days the after-treatment of the cases was made very much more difficult, and the convalescence very much slower, by the method of treating the pedicle. While McDowell had used the ligature, dropping the pedicle, and had done so successfully, others seemed to be afraid of following his example. The great doubt was as to what would become of the piece outside of the ligature. This, it was feared, would die, and poison the patient. Many of the deaths in the early cases were attributed to this cause. To overcome this difficulty, various plans were suggested. Baker Brown used the cautery, and, as Mr. Tait pointed out, had he lived, no doubt abdominal surgery would have been advanced many years; for, although we cannot help acknowledging an immense debt as due to Sir Spencer Wells, still we cannot deny that he kept back ovariotomy and abdominal surgery by his energetic advocacy and use of the clamp. His plan was to clamp the pedicle, leaving it on the outside, the abdomen being closed tightly around it, the clamp preventing it from falling in.

Dr. Thomas was a bold and brilliant operator, a great diagnostician, and full of invention and resources. His record after these early years is well known, though he came a little too late to reap the full advantages of modern abdominal surgery. To my association with Dr. Thomas in those early days, I must attribute my interest in this branch of medicine, and, to a great extent, my success. To no man, living or dead, do I owe more than to him. In fact, had it not been for Dr. Thomas, I should not have held my present positions, as it was by his influence that I became Dr. White's successor and a resident of Buffalo. Dr. Sims, although I knew him well and have seen him do some plastic work, I never had the pleasure of seeing open an abdomen. Dr. Peaslee I also knew well, but never saw him operate.

In those days the New York Obstetrical Society was the scene of many exceedingly interesting discussions. Abdominal surgery and gynecology were making rapid strides in advance. Sims, Peaslee, Thomas, and Emmet were the four men who have done more for gynecology than any Americans who have ever lived. They were then making rapid advances, and in the obstetrical society the new ideas were proposed and weighed and discussed, to be afterwards tested at the bedside and on the operating table, and the results reported back to the society. I was secretary for a number of years, and had the great advantage of being obliged to take down these discussions. I am sure that this was of great benefit to me, as it fixed in my mind a great many facts which I probably should not otherwise have learned.

Besides these greater lights, Noeggerath, whose name is well known as the discoverer of latent gonorrhea; Jacobi, still a nestor in the profession; besides some of the younger men, who have since made name and fame, were active members of the society.

Buffalo took a prominent part in the early days of abdominal surgery. Drs. James P. White and Julius F. Miner were both pioneers. Dr. White probably did a hundred ovariotomies during his life, about 60 per cent. of which recovered, as far as I can learn. Dr. Miner never did so many, but he originated a principle which has made his name to be mentioned wherever the history of ovariotomy has been spoken of—he originated the idea of enucleation. This I had seen done by Dr. Thomas, but had never practised until I did my first ovariotomy in Buffalo.

My first case was done in Hartford, Connecticut, in 1879. The patient was a poor negress. I had to pay the nurse myself, and, as she lived four miles in the country, in a poor little farmhouse, I had to hire a horse each time I made a visit. As you can readily imagine, I did not make a fortune immediately out of the case. Still its effects on my future were greater than were at first apparent. The event was a great one, and my friend, Dr. Munde, came all the way from New York to assist me. He had never operated himself, nor had anyone else present ever seen an ovariotomy. I found a dermoid cyst so adherent that I could not get it all out. I therefore cut off all I could get loose, and sewed the edges of the remaining portion to the edges of the abdominal wound. Two glass drainage tubes were used, one being put into the sac and the other into the abdominal cavity. The patient convalesced very slowly, and required many visits. I estimated that the case cost me $50. Still it paid, for it gave me experience, and allowed me to say that I was an operator—great advantages when the call came to go to Buffalo.

To illustrate the fear which the early ovariotomists had of the peritoneum, I remember very distinctly a case which came to me a number of years ago. She had a large fibroid tumor and a tremendous ventral hernia. She told me that she had had an ovarian tumor, which had been removed by Dr. Miner, the first successful operation that he had ever done. She showed me a copy of an account of the operation, published in the BUFFALO MEDICAL JOURNAL at that time. In this article, Dr. Miner attributes his success to the fact that he did not pass his stitches through the peritoneum, but only through the skin and fat. This, while it does not explain the success of the operation, certainly explains the ventral hernia. I removed the fibroid, and sewed up the hernia, and sent the woman home cured.

Thus far I have spoken only of ovariotomy; but it is quite natural that the opening of the abdomen for the removal of ovarian tumors should have led to the same procedure for other purposes. In 1876, Dr. Robert Battey, of Rome, Ga., read a paper before the American Gynecological Society, on The extirpation of the functionally active ovaries. He had performed his first operation in August, 1872. In 1879, Mr. Lawson Tait announced that he had done a similar operation, claiming priority over Battey. Prof. Hegar, of Freiburg, in Germany, published in 1878, a paper on The castration of women, his first case having antedated Battey's by a month. After the publication of these papers, the indications for opening the abdomen were very quickly widened, and the operation took firm hold upon the profession, being performed by operators all over the world; and at that time we may say that abdominal surgery, other than ovariotomy, had its origin.

I first removed the ovaries, March 11, 1880, in Hartford, Conn., for a fibroid tumor. The first operation for the removal of the ovaries which was done in western New York, was performed by the late Dr. G. C. Clark, of Niagara Falls, 1882. I had the pleasure of assisting him; the operation was perfectly successful.

My first operation in Buffalo for the removal of the ovaries was in November, 1883. On March 4, 1884, I did my first resection of intestine; likewise the first that was done in Buffalo. In October of the same year, I removed a large fibroid tumor by supra-vaginal hysterectomy with the clamp. The woman is still living.

Although I did many operations for the removal of ovaries and fibroids from that time on, it was not until February, 1888, that I first removed pus tubes. After this, the indications for operations and the number of cases increased rapidly; but I did not meet with a case of extrauterine pregnancy until 1890. I operated on four during that year. As I was almost the only operator practising abdominal surgery in Buffalo then, these were doubtless the first operations of their kind which were done there.

We thus see that abdominal surgery is of very recent development, the greatest growth and extension of the operation having taken place in the decade between 1880 and 1890. It may now be said to be nearly perfected, and, except in operations on the gall-bladder and the stomach, we cannot look forward to many more advances.

What has made possible the great successes of modern abdominal surgery? Two things will at once come to the mind of each of you—anesthesia and antisepsis. Without these there could have been no development. Although the early operations were done without anesthesia, the operations now undertaken would be impossible under similar conditions.

Nor is antisepsis—or, perhaps, more strictly speaking, asepsis—any less important. The mortality rates of the pioneers are often frightful to contemplate; and only where life was directly threatened, as in ovarian cystic disease, were operations warranted. So recently as 1880, the writer collected all the known cases of oophorectomy—150, with a mortality of 20 per cent.; and in 1884, Bigelow collected 359 hysterectomies for fibroids, with 58 per cent. mortality. Now all this is changed, and we open the abdomen, even in comparatively simple diseases, with perfect confidence in the result, as far at least as sepsis goes. So much has been accomplished by Lister, Pasteur, and their co-workers.

But, after all, is it not to the American workers that a very large share of the mead of praise is due? Who have done more than McDowell, Nathan Smith, the Atlees, Kimball, Miner, Sims, Peaslee, Thomas, Robbs, Battey, Sands, McBurney, and Bull—to say nothing of the men of our own day, who have improved, extended, and perfected the work of their predecessors? Certainly America has a right to be proud of the credit of originating and perfecting this important branch of surgical work. Not only did ovariotomy originate here, but hysterectomy for fibroids was first done by Kimball. Peaslee and Sims originated drainage; Battey first removed diseased ovaries; Willard Parker did the first operation for disease around the appendix; while Sands, McBurney, Senn, and Weir were the pioneers in appendectomy. Bull did the first operation for bullet-wound of the intestines; and Rogers was the first to advocate the operation for ruptured tubal pregnancy. Kimball's lead in removing fibroids was followed by many, and was so perfected by the work of Stimson, Polk, Baer, Pryor, and others, that it is now known as the "American operation." Robbs was the first to do the modern operation of cholecystectomy, while the genius of Sims had a most important influence on advancing this particular branch of surgery.

But I need not add to the list. It is recent history and familiar to all students of contemporary literature.

When we look back and see what has been accomplished, it seems almost miraculous—all fear of the peritoneum gone; sepsis nearly banished, and scarcely an organ in the abdomen which has not been successfully attacked and removed. Liver, gall-bladder, spleen, stomach, intestines, kidney, uterus, tubes, ovaries, bladder—all have yielded to the surgeon's knife, and their possessors relieved of serious or fatal diseases. It is a proud record. Little did McDowell think, when he took up the knife to make his first abdominal section, to what it would lead, and of the years of agony which would be relieved and the thousands of lives saved. All honor to the men who have done this work. Their names should stand higher in the roll of fame than those of generals and conquerors. They have worked to relieve pain and suffering, and to save life, while the soldiers accomplished their ends only through the infliction of measureless agony, and the sacrificing of countless lives.

31 Allen Street.

